# The Impact of Poor Sleep Quality on Cardiovascular Risk Factors and Quality of Life

**DOI:** 10.7759/cureus.77397

**Published:** 2025-01-13

**Authors:** Chidinma J Onyegbule, Chioma G Muoghalu, Cosmas C Ofoegbu, Franklin Ezeorah

**Affiliations:** 1 Family Medicine, Alex Ekwueme Federal University Teaching Hospital, Abakiliki, NGA; 2 Medicine, University of Galway, Galway, IRL; 3 Health Sciences, Central Washington College Enugu, Enugu, NGA; 4 Community and Family Medicine, Al Lith General Hospital, Al Lith, SAU; 5 Psychology, University of East London, London, GBR; 6 Psychology, California Southern University, Costa Mesa, USA

**Keywords:** cardiovascular disease, hypertension, obesity, poor sleep quality, quality of life, sleep

## Abstract

This review article examines the impact of poor sleep quality on cardiovascular risk factors and quality of life. Insufficient sleep and disturbances in sleep have been linked to a variety of negative health outcomes, including an increased risk of cardiovascular disease, elevated blood pressure, obesity, and a reduced quality of life. This review explores the available evidence connecting poor sleep with these health conditions, analyzing the underlying mechanisms and pathways involved. Additionally, the challenges posed by night work, which can contribute to poor sleep and subsequent health problems, are discussed. The review also discusses evidence-based strategies for improving sleep quality, encompassing sleep hygiene practices, bright light therapy, cognitive behavioral therapy for insomnia, pharmacological interventions, and emerging digital health solutions. The aim is to analyze current research and emphasize the crucial role of prioritizing sleep quality in maintaining both cardiovascular health and quality of life.

## Introduction and background

Sleep is a cyclic physiological state characterized by decreased responsiveness, from which individuals awaken spontaneously [1]. Contrary to popular belief, the brain is often more active during sleep than during wakefulness, involving considerable physiological activity [1]. Human sleep consists of two main types: non-rapid eye movement (NREM) sleep and rapid eye movement (REM) sleep [2]. These phases are associated with distinct levels of arousal, autonomic responses, brain activity, and muscle tone, occurring alternately in cycles of approximately 90 minutes throughout the night [1,3,4]. NREM sleep is linked to increasing sleep depth and is further classified into four stages. Stages 3 and 4 are combined to form slow-wave sleep, which is believed to be the most restorative type of sleep and typically occurs in the first one-third of the night [2]. REM sleep is characterized by vivid dreams, loss of muscle tone, and REMs. Generally, the initial part of the night consists mainly of NREM sleep, while REM sleep occurs in the second half. The ultradian rhythm regulates the timing and duration of these sleep states, describing the alternation of NREM and REM sleep throughout the night [1].

Sleep has a positive impact on human health and well-being, and a sufficient amount is necessary for individuals to remain alert and function adequately throughout the day [5]. It plays a critical role in the proper functioning of various body systems, including the immune, hormonal, and cardiovascular systems. Sleep is considered normal and healthy if it meets several criteria: it should be of sufficient duration, good quality, appropriately timed, and regular [2]. Adequate sleep promotes optimal restoration of the body and tissue and increases the production of anabolic hormones like growth hormone, prolactin, testosterone, and luteinizing hormones while simultaneously reducing the levels of catabolic hormones such as cortisol. Additionally, sleep conserves energy by lowering blood pressure and slowing metabolism [6]. On average, adults require approximately 8 hours of sleep, regardless of cultural or environmental factors. Insufficient sleep can lead to exhaustion, irritability, reduced concentration, and higher rates of cancer, depression, and anxiety [6]. People typically spend about one-third of their lives sleeping. Those who get enough sleep tend to experience more energy, better cognitive function, a healthier immune system, improved memory, increased alertness, and better overall performance during the day [7].

The two main neurobiological mechanisms regulating sleep are the circadian and homeostatic mechanisms [1]. The circadian rhythm governs periods of sleep and wakefulness based on the cycles of light and dark. In contrast, the homeostatic mechanism increases sleep drive after a sleep debt has accumulated, aiding in rejuvenation and restoring alertness in both body and mind [1]. The hormonal regulation of sleep is controlled by melatonin, a hormone secreted by the pineal gland in the brain. Melatonin secretion is stimulated by darkness and suppressed by light, resulting in maximum serum concentration at night and an increased desire to sleep [8]. According to the National Institute of Health, sleep deficiency occurs when individuals do not receive enough sleep (sleep deprivation), have sleep habits that are out of sync with the body's natural circadian rhythm, or experience diminished sleep quality or quantity due to sleep disorders or external factors [9].

Sleep disorders encompass a variety of syndromes characterized by disturbances in the amount, quality, or timing of sleep or associated behavioral and physiological conditions [10]. These disorders may manifest in several ways, such as sleep deprivation, difficulty maintaining sleep (as seen in insomnia or sleep fragmentation), and events occurring during sleep, like sleep apnea and restless leg syndrome. The prevalence of sleep disorders is significant, often leading to reduced sleep duration [2,11]. A study done by McArdle et al. suggests that at least one sleep disorder was present in 41.0% of females and 42.3% of males [12]. A study from South Korea highlighted that disruptions in circadian rhythms may contribute to sleep disorders [11]. Furthermore, recent research indicates that circadian misalignment can impair metabolic and cardiovascular functions [13,14]. Circadian rhythms are natural sleep-wake cycles that align with the light-dark cycle of day and night [15]. Circadian rhythms possess three main characteristics: they are endogenous, resistant to abrupt changes, and slow to adapt to changing conditions [15].

The central nervous system (CNS) acts as the primary synchronizer of circadian rhythms, coordinating pacemakers in the brain and peripheral tissues via signals from the suprachiasmatic nucleus (SCN) in the hypothalamus [16]. The SCN promotes increased alertness during the day and sleep propensity at night [14]. Neurons in the hypothalamus regulate the sleep-wake cycle by inhibiting arousal systems, allowing for sleep [2]. The light-dark cycle serves as the most critical synchronizer for the central pacemaker. The SCN receives direct impulses from the retina via specialized nerve cells known as brightness detectors [2]. This light input signals the pineal gland to regulate melatonin secretion, which synchronizes the circadian rhythm with the external environment. Any disturbances to this well-regulated rhythm can lead to various sleep disorders [2]. Changes in the sleep-wake cycle can disrupt circadian synchronization. Repeated desynchronization and resynchronization of circadian rhythms have both long-term and short-term consequences [17,18]. Short-term consequences of sleep deprivation include impaired attention and concentration, decreased quality of life (QOL), increased absenteeism, reduced productivity, and a higher risk of accidents [19]. Long-term consequences can consist of coronary artery disease, heart failure, high blood pressure, obesity, type 2 diabetes mellitus (T2DM), stroke, memory impairment, and depression [19].

The aim of this study is to investigate the negative effects of poor sleep quality on cardiovascular risk factors and overall QOL. It will examine the relationships between insufficient or disrupted sleep and various health outcomes, including cardiovascular disease (CVD), blood pressure, obesity, and a reduced QOL, in addition to other health issues like metabolic syndrome, T2DM, gastrointestinal diseases, psychiatric disorders, certain cancers, menstrual cycle disruptions, and increased miscarriage. Additionally, the study will explore the specific impact of night work on sleep quality and its subsequent health consequences, taking into account the challenges faced by night workers in maintaining healthy sleep patterns. The research will synthesize current evidence-based strategies for improving sleep quality, including sleep hygiene practices, bright light therapy, cognitive behavioral therapy for insomnia (CBT-I), pharmacological interventions, and digital health solutions, focusing on their effectiveness for at-risk populations. The crucial role of prioritizing sleep quality in maintaining both cardiovascular health and overall well-being, particularly for individuals with sleep disorders and those who work night shifts, will also be emphasized.

## Review

Sleep quality and cardiovascular health

CVDs account for approximately one-third of all deaths worldwide [20]. Poor sleep quality and quantity, whether due to sleep disorders or inappropriate sleep patterns, are linked to various risk factors for CVDs, including hypertension, obesity, diabetes, and dyslipidemia [9]. Sleep deprivation triggers inflammatory processes and activates the sympathetic nervous system (SNS), which can raise blood pressure and impair glucose tolerance. These factors collectively increase the risk of developing CVDs and diabetes [21]. Research indicates that circadian misalignment is associated with cardiometabolic changes and a heightened risk of metabolic syndrome and CVDs [22]. CNS, which regulates the circadian pacemaker, also triggers melatonin secretion during the night. However, exposure to light during night shifts is detected by retinal receptors, which send signals to the suprachiasmatic nuclei and the pineal gland, leading to suppressed melatonin production [23]. Several studies have demonstrated that higher melatonin levels are associated with lower risks of hypertension, T2DM, and the incidence of myocardial infarction, while reduced melatonin level causes an increased risk of CVDs [23-26]. Additionally, sleep plays a regulatory role in glucose metabolism and the hormones that control appetite [27]. Consequently, recurrent exposure to short sleep durations and poor sleep quality increases the risk of CVDs.

Sleep quality and hypertension

The etiology of primary hypertension is unknown, but several factors contribute to its development, including obesity, increased salt intake, and a positive family history [28-30]. Hypertension affects nearly two-thirds of adults and is the most common risk factor for CVDs [31]. Changes in blood pressure are normally followed by feedback mechanisms that help return blood pressure to normal levels [32]. Hypertension occurs when the body is unable to compensate for these changes [32]. Normal blood pressure is maintained by cardiac output and total peripheral resistance (TPR) [32]. Activation of the heart, kidneys, and peripheral blood vessels by the SNS leads to an increase in cardiac output, TPR, and fluid retention, all of which are involved in the development and maintenance of hypertension [31]. Poor sleep quality has been linked to disruption in this feedback mechanism [31].

The renal SNS plays a major role in the development and maintenance of hypertension. This occurs through either efferent or afferent pathways [31]. In the efferent pathway, signals from the SNS stimulate the kidneys to release renin, activating the renin-angiotensin-aldosterone system [31]. Over time, this leads to salt and water retention, which increases circulating blood volume and consequently raises blood pressure. Additionally, the efferent pathway reduces blood flow to the kidneys. The reduced blood flow signals the SNS to increase sympathetic activity, maintaining elevated blood pressure [31]. Under normal sleep conditions, the parasympathetic nervous system is activated and dominant, leading to a decrease in the activity of the SNS. This results in a reduction in the synthesis of catecholamines, such as adrenaline and noradrenaline with a subsequent reduction in blood pressure [33]. Conversely, sleep deprivation acts as a stressor on the body, activating the sympathetic system. This activates the renin-angiotensin-aldosterone system and increases the synthesis of central catecholamines, resulting in blood vessel constriction and elevated blood pressure, which can lead to hypertension [33]. There is evidence supporting the relationship between poor sleep quality and short sleep duration with SNS activation, resulting in elevated blood pressure [27].

Another theory suggests that disruptions in the circadian rhythm may contribute to hypertension through various mechanisms, including impaired endothelial function resulting from decreased nitric oxide levels [34,35]. Poor sleep quality has been linked to a non-dipping blood pressure pattern, where nighttime blood pressure fails to drop significantly. This shift from a dipping pattern, characterized by a drop of more than 10% in average mean arterial blood pressure, to a non-dipping pattern has been associated with increased cardiovascular risks [35,36]. Individuals classified as "non-dippers" typically exhibit heightened SNS activity, and reduced parasympathetic activity which leads to higher nocturnal blood pressure [37]. Night-time BP has been shown to be a better predictor of cardiovascular risk than daytime BP [35]. Nondipping of the blood pressure is related to target organ damage in patients with hypertension and is a predictor of CVD morbidity and mortality [35-37].

Calhoun et al. observed a 21% increase in CVD mortality for subjects who had a 10 mmHg increase in systolic nocturnal BP [35,38]. Further, poor sleep quality has been associated with pre-hypertension in healthy adolescents and a higher prevalence of hypertension in adults; however, the evidence connecting poor sleep to the development of hypertension is inconsistent, showing varying associations across different regions [37]. A meta-analysis by Kenneth et al. examined studies conducted over 50 years across multiple continents and found a positive association between poor sleep and hypertension, although the higher average blood pressure scores among individuals with poor sleep were statistically insignificant [37]. Conversely, a cross-sectional study in the United States by Bansil et al., based on data from the National Health and Nutrition Examination Survey (NHANES), found no relationship between sleep disorders and hypertension when short or poor sleep was not present. However, individuals experiencing both sleep disorders and short sleep duration were more than twice as likely to have hypertension compared to those without sleep-related issues [39].

In a prospective cohort study in Colombia involving 323 women, Aggarwal et al. identified an association between poor sleep quality and elevated systolic blood pressure [40]. Similarly, Meng et al. found that hypertensive subjects in their study in China were more likely to experience shorter sleep duration [41]. Additionally, a cross-sectional study by Lu et al. in 2013 on Chinese adult men examined the individual and combined effects of three aspects of sleep on hypertension. They observed a correlation between poor sleep quality and short sleep duration with an increased prevalence of hypertension, although this finding was not statistically significant [42]. In conclusion, while there is some evidence suggesting a link between poor sleep quality and hypertension, the relationship appears to be complex and varies across different populations. Further research is needed to clarify these associations and understand the underlying mechanisms involved.

Sleep quality and obesity

Obesity, defined as a body mass index (BMI) greater than 30 kg/m², represents excess body weight for height or an abnormal accumulation of body fat, typically 20% above normal, which can adversely affect health [43,44]. It is a global public health issue, with one in 10 adults classified as obese [44]. This condition is associated with an increased risk of various diseases, CVDs, T2DM, obstructive sleep apnea, certain cancers (such as endometrial, colorectal, and breast cancers), osteoarthritis, asthma, infertility, and depression [45-47]. Sleep plays a crucial role in regulating glucose metabolism and appetite-related hormones [27]. Individuals who experience insufficient sleep tend to have elevated levels of ghrelin, a hormone that stimulates appetite, and reduced levels of leptin, which suppresses appetite [48]. Ghrelin promotes food intake while decreasing fat utilization [48]. Consequently, sleep deprivation can lead to increased food consumption, as the body seeks to compensate for the energy demands of extended wakefulness [49]. This physiological response may contribute to overeating. Moreover, orexin/hypocretin neurons, which are vital to sleep-wakefulness and feeding regulation, show increased activity during sleep loss [49]. These orexigenic neurons influence the homeostatic feeding center in the arcuate nucleus of the hypothalamus. Leptin and ghrelin, as peripheral signals, interact with this area to modulate food intake [22]. The desynchronization of circadian rhythms related to these hormones can create an imbalance between energy intake and expenditure, leading to weight gain [48].

Decreased REM sleep is commonly observed in individuals with short sleep duration and is proposed as a mechanism linking insufficient sleep to weight gain. Additionally, sleep deprivation may disrupt energy balance by reducing both exercise and non-exercise energy expenditure [50]. Lower leptin levels following sleep deprivation can negatively impact caloric intake and energy expenditure [50]. Increased sleepiness and fatigue often lead to sedentary behavior, further decreasing exercise-related energy expenditure [6]. Stress, which adversely affects sleep quality and duration, can exacerbate sedentary behavior and decrease insulin sensitivity and glucose tolerance, potentially resulting in increased food intake and weight gain [6]. Furthermore, inadequate sleep reduces melatonin levels, leading to metabolic dysfunction and increased insulin resistance, which can ultimately contribute to obesity [51]. Melatonin regulates the circadian rhythms of various metabolic hormones, including insulin, cortisol, and leptin. However, light exposure can suppress melatonin secretion, disrupting the diurnal variation of these hormones and impairing energy metabolism [52].

Research supports the association between sleep quality and obesity. For instance, a study by Logue et al. found that reduced sleep duration and poor sleep quality correlated with obesity [53]. Kristicevic et al. reported that poor sleep quality was linked to an increased risk of being overweight or obese among Croatian young adults [54]. Similarly, Peltzer et al. identified an association between poor sleep quality and increased waist circumference in university students from 26 different countries, though no association with BMI was found [55]. In a Swedish population-based study, Haglow et al. noted an independent inverse relationship between measured sleep duration and waist circumference [56]. Conversely, some studies, such as those by Lee et al. and Abdussalam et al., reported no significant correlation between sleep duration and BMI [57,58]. Bidulescu et al. found an association between obesity and overall sleep quality scores in Atlanta [59].

Furthermore, an Ethiopian study by Berhanu et al. indicated that obese individuals were more likely to experience poor sleep quality compared to those with a normal BMI [60]. A study conducted in Ilorin, Nigeria, by Shittu et al. demonstrated a strong statistical association between obesity and measured sleep scores [61]. While epidemiological evidence suggests an association between increased sleep duration and reduced obesity, these studies do not establish a causal relationship [62]. In summary, while there is a notable association between sleep quality and obesity, the relationship is complex and influenced by various physiological and environmental factors. Addressing sleep quality may be a critical component in tackling obesity and its related health risks. Further research is necessary to explore the causal pathways and develop effective interventions.

Sleep quality and QOL

The concept of QOL was introduced into medical literature in the 1960s and has become increasingly relevant in recent decades. In 1975, QOL was established as a keyword in medical literature databases [63]. By the 1990s, assessments of QOL started to focus on the individual’s emotions and inner life. Researchers began to explore not only the objective quantitative parameters related to QOL but also its subjective aspects, particularly an individual’s sense of satisfaction [64]. In 1997, the World Health Organization (WHO) defined QOL as an individual's perception of their position in life considering the culture and value systems in which they live, along with their goals, expectations, standards, and concerns [64,65]. Understanding QOL is crucial for grasping an individual's well-being, as it encompasses physical, mental, and social aspects of life [66]. Specifically, health-related quality of life (HRQOL) refers to an individual's general health and well-being concerning symptoms and functioning, reflecting how they value a particular state of health [67]. HRQOL consists of four dimensions: physical and motor skills, mental state, social and economic conditions, and somatic perception (related to symptoms such as pain). This concept emphasizes the need to distinguish between an objective state of health and the subjective experience of the patient [64].

Sleep disorders can lead to morbidity and mortality, as well as decreased functional capacity and QOL [65]. In addition, poor sleep health has been linked to negative mental health outcomes [67]. Sleep disorders may also result in the abuse of sleep medications, further contributing to a decline in QOL [68]. Therefore, the impact of sleep disorders is vital when evaluating the QOL within any population [5]. Several studies have linked poor sleep quality to impaired QOL. Palhares et al. conducted a cross-sectional study involving 264 nursing professionals in Brazil, demonstrating a close relationship between sleep quality and QOL, where alterations in sleep were associated with impaired QOL [65]. A study by Roeser et al. examined the relationship between sleep quality and HRQOL among adolescents in Berlin and found a significant positive correlation between these two factors [69]. In their work with nurses in various hospitals in Iran, Zamanian et al. noted a positive relationship between QOL and sleep quality; however, they could not establish that QOL is predictive of sleep quality [5]. A study by Lo et al. in China showed that participants with poor sleep quality had lower scores across all domains of QOL, and this was statistically significant [70]. Shao et al. revealed that nurses who reported illnesses such as gastrointestinal diseases, CVDs, asthma, allergies, and joint or muscle pain had higher sleep quality scores that correlated with lower QOL scores [71].

A South Korean study involving 2,238 working women from 2011 to 2013, utilizing data from the Korea Health Panel, demonstrated that women working night shifts had lower QOL scores compared to those who worked during the day. This study also indicated that daytime workers had better physical health and work ability than their night-shift counterparts [66]. Similarly, Selvi et al. found that night workers in a hospital in Turkey had poorer scores in some QOL domains compared to day workers, which was statistically significant [67]. Nena et al. conducted a cross-sectional study on healthcare workers at a tertiary hospital in Greece, which showed that shift work could impair an employee’s sleep characteristics and overall QOL [72]. Another study by Selvi et al. assessed the impact of shift work on the psychological state and QOL of health workers, highlighting that the QOL of shift workers was adversely affected [67]. Boughattas et al. in their cross-sectional study on Tunisian nurses showed a better QOL among nurses working during the day compared with those on night shift [73]. Sleep quality significantly impacts QOL, with poor sleep consistently associated with reduced well-being across multiple domains. Interventions aimed at improving sleep quality are crucial for enhancing overall health and well-being.

Other health problems associated with poor sleep and night work

Poor nighttime sleep disrupts circadian rhythms, which are crucial for normal daily metabolic functions [11]. These rhythms help regulate energy expenditure and hormones involved in energy metabolism, such as leptin, ghrelin, insulin, and melatonin. Disruption of sleep and circadian rhythms can contribute to insulin resistance, impaired glucose regulation, and the development of T2DM [74]. Some studies have indicated that night workers experience impaired glucose tolerance, increased insulin resistance at night, and a higher prevalence of T2DM, with this risk increasing alongside the number of years worked [23]. The exact mechanism through which night work causes metabolic dysregulation remains unclear, but it may involve hormonal alterations and increased sympathetic drive, leading to decreased insulin sensitivity and insufficient beta-cell compensation [74]. Furthermore, altered melatonin synthesis due to circadian misalignment may also play a role [18]. Some studies have demonstrated that higher melatonin levels are associated with a reduced risk of diabetes [25,26]. Melatonin receptors have been identified on pancreatic islet beta-cells, indicating that melatonin can control insulin secretion [25].

Jet lag is a common issue for night workers, contributing significantly to the development of metabolic syndrome [75]. It induces changes in cholesterol levels and disrupts normal digestive processes [75]. Research has shown that social jet lag can lead to increased body weight gain due to a higher consumption of fast foods [75]. Metabolic syndrome is an independent risk factor for coronary heart disease, and there is a documented increase in coronary heart disease incidents among night duty workers, likely explained by a higher prevalence of metabolic syndrome [76]. In a study by Baek et al. utilizing data from the National Survey of Midlife Development in the United States (MIDUS), a significant correlation was found between sleep quality and HbA1c levels. Additionally, positive correlations were observed between sleep quality and total cholesterol and triglyceride levels [51]. A Nigerian study by Adeoye et al. among 256 hospital patients in Ibadan found that the overall prevalence of metabolic syndrome was 24.2%, with women exhibiting a higher prevalence [76]. Night work is also known to increase the risk of gastrointestinal diseases.

Studies indicate that shift workers experience functional digestive disorders more frequently and are more susceptible to various gastrointestinal illnesses, including digestive ulcers and gastroesophageal reflux [77]. These digestive issues are primarily attributed to misalignments between meal times and normal circadian rhythms governing gastrointestinal functions, such as gastric bile and pancreatic secretions, enzymatic activity, intestinal motility, and nutrient absorption rates [77]. Moreover, night work may contribute to several psychiatric disorders due to circadian misalignment, sleep deprivation, and melatonin suppression [75]. There is a noted reduction in serotonin secretion, a neurotransmitter that promotes good mood and regulates sleep, among shift workers, which can lead to moodiness [78]. Irregular and continuously changing shifts may cause genetic alterations and uncontrolled expression of genes, contributing to disorders such as bipolar disorder, major depression, and schizophrenia [75].

Some studies also suggest that night work increases the risk of developing certain cancers. The International Agency for Research on Cancer, part of the WHO, has classified night work as having a potential carcinogenic effect on humans due to its disruption of biological circadian rhythms [77]. The mechanisms through which circadian disruption may promote malignant tumors are complex and multifactorial. This disruption can alter the rhythmicity of various genes in breast tissues, affecting normal tissue structure and biology, and potentially leading to breast cancer [79]. The endocrine changes induced by circadian disruption and melatonin suppression from light exposure may target endocrine-responsive tissues, such as breasts in women and the prostate in men. Melatonin plays a role in inhibiting cancer development and growth, as well as enhancing immune function [21,80]. Repeated internal desynchronization may impair circadian regulation of cell cycles, favoring uncontrolled growth. Sleep deprivation can also suppress immune surveillance, which may allow the establishment and/or growth of malignant clones [23]. The sleep disruptions caused by night work are associated with elevated prostate-specific antigen (PSA) levels, indicating an increased risk of developing prostate cancer among night workers [23,40]. In addition, other mechanisms like inflammatory processes, or changes in the autonomous tone, could possibly mediate the link between sleep deficiency and cancer risk [21].

The menstrual cycle can be disrupted in night workers, potentially due to the influence of circadian rhythms. Night workers experience a higher incidence of altered menstrual cycles, premenstrual syndrome, and menstrual pain [23]. Additionally, the rates of miscarriage, impaired fetal development, preterm birth, and low birth weight are also more common among night workers [23]. Sleep deprivation associated with night work affects perception and decision-making abilities, increasing the likelihood of accidents and injuries [81]. Poor quality sleep results in increased sleepiness while working, as demonstrated in various studies. This sleepiness is often responsible for accidents, decreased interest, anxiety, irritability, reduced efficiency, and heightened stress [81]. A study by Fadeyi et al. on predictors of night work disorders found that the prevalence of needle prick injuries was higher among nurses on night duty.

Additionally, there is evidence suggesting that night shift workers may struggle to respond appropriately to emergencies [82,83]. Moreover, individuals with chronic illnesses may find it more challenging to manage their symptoms and disease progression when working night shifts. Some medications vary in effectiveness throughout the circadian cycle, making it harder to determine the correct dosage and timing when working nights. Night work can also interfere with treatment regimens that require maintaining regular sleep patterns [80]. The effects of night work are significant and are often more pronounced in women, likely because women typically manage greater family responsibilities. Women in night work may experience higher levels of work-family role conflict and tend to have lower tolerance for shift work. They also report experiencing more fatigue and sleepiness compared to their male counterparts [66].

Improving sleep quality: A multifaceted approach for at-risk populations

Sleep is an essential aspect of overall health and well-being. However, many individuals, particularly vulnerable populations such as night workers and those with sleep disorders, face challenges in maintaining high-quality sleep. Improving sleep quality is critical not only for enhancing daily functioning but also for long-term health outcomes.

Evidence-based strategies to improve sleep quality

Several strategies can significantly improve sleep quality, particularly for individuals who are at risk, such as night shift workers or those with sleep disorders like insomnia or sleep apnea. One of the most effective approaches for vulnerable populations is creating a consistent sleep environment and routine. Research has shown that sleep hygiene practices, such as maintaining a consistent sleep schedule, creating a quiet, dark environment, and avoiding caffeine or heavy meals before bedtime are essential for improving sleep quality [84]. Additionally, a cool room temperature and the use of earplugs or white noise machines can also enhance the sleep environment, especially for individuals working at night. Night shift workers face unique challenges, such as misalignment between their circadian rhythms and their work schedule, leading to poor sleep quality and increased risk for chronic conditions like CVD [11]. One effective strategy for mitigating these risks is the use of bright light therapy. Exposure to bright light during the night shift and minimizing light exposure during the day help realign the body's internal clock [85]. Moreover, napping during breaks in a well-designed sleep environment can provide short-term relief and improve alertness [85].

CBT-I and pharmacological interventions

For individuals with insomnia, CBT-I has become a gold standard in non-pharmacological interventions. CBT-I targets the thoughts and behaviors that disrupt sleep, including poor sleep hygiene and maladaptive beliefs about sleep. Numerous studies have shown CBT-I to be highly effective, with benefits that can last months or even years [86]. In addition to CBT-I, pharmacological treatments such as benzodiazepines, selective serotonin reuptake inhibitors (SSRIs), and melatonin receptor agonists are commonly prescribed for sleep disorders; however, these treatments often come with side effects and risks of dependency, particularly with long-term use [87]. A study by Roth et al. suggested that low-dose doxepin could be helpful in treating insomnia with minimal risk, although their efficacy can vary depending on the individual [88].

Digital health interventions and technology’s role in sleep

Digital health interventions have risen significantly in recent years, offering new tools for individuals seeking to improve their sleep quality. Apps and wearable devices designed to monitor sleep patterns and provide personalized feedback are becoming more popular. These technologies can help users track variables like sleep duration, sleep stages, and even environmental factors such as room temperature and light exposure. Recent studies, such as a study by Mahmud et al., demonstrated that sleep-tracking apps and interventions that provide guided sleep improvement programs have been shown to enhance sleep quality in both healthy individuals and those with sleep disorders [89]. Artificial intelligence (AI)-powered platforms are also being developed to help diagnose and manage sleep conditions, offering a more personalized approach. For example, AI-based virtual sleep coaches, integrated into smartphones or wearable devices, offer real-time suggestions and adjustments based on data collected throughout the night. Research by Bandyopadhyay et al. highlighted the potential of AI-driven sleep coaching as an effective intervention for improving sleep patterns in individuals with chronic insomnia [90].

Impact of artificial light and sleep in aging populations

The impact of artificial light on sleep has become a major concern in modern society. Exposure to blue light from screens, especially before bedtime, has been linked to disruptions in circadian rhythms and poor sleep quality [91]. As the use of digital devices has skyrocketed, this form of light exposure is now a significant factor in sleep disturbances. Strategies like reducing screen time in the evening and using blue-light-blocking glasses have been recommended to help mitigate these effects. In aging populations, sleep quality tends to decline due to various factors such as changes in circadian rhythms and the presence of chronic health conditions. A study by Lee et al. found that older adults often experience lighter sleep and more frequent awakenings [92]. Interventions aimed at improving sleep in the elderly often focus on behavioral strategies, pharmacological treatments, and managing comorbid conditions [93]. Furthermore, social and environmental factors such as increased daytime activity and reduced nighttime environmental stressors have been shown to improve sleep quality among older adults [93].

## Conclusions

In conclusion, the relationship between poor sleep quality and cardiovascular health, as well as overall well-being, is complex and multifaceted. Evidence suggests that factors such as inadequate sleep duration, disrupted sleep patterns, and the unique challenges faced by night workers can increase the risks of CVDs, hypertension, obesity, and poor QOL, in addition to other health issues like gastrointestinal diseases, psychiatric disorders, certain cancers, menstrual cycle disruptions, and increased miscarriages. However, findings regarding hypertension are not consistently applicable across different populations.

The mechanisms underlying these associations involve various physiological and psychological pathways. Addressing poor sleep quality remains a crucial public health priority. It is crucial for overall health, particularly for vulnerable populations such as night workers and individuals with sleep disorders. Evidence-based strategies, such as maintaining good sleep hygiene, using bright light therapy, and employing interventions like CBT-I, offer effective ways to enhance sleep. Recent advancements in digital health interventions and AI technologies provide innovative solutions to monitor and improve sleep. Additionally, the impact of artificial light exposure and the unique challenges of aging populations require targeted approaches to ensure better sleep quality for all. Future research should focus on developing tailored interventions to improve sleep hygiene, particularly for those facing specific challenges, such as night workers. These efforts could ultimately lead to enhanced cardiovascular health and an improved overall QOL for individuals and communities.
